# Electronic Structure and Thermal Properties of a Cubic Laves Compound NaAg_2_ Featuring a [Ag_4_]^2–^ Pyrochlore Network

**DOI:** 10.1021/acs.inorgchem.5c05773

**Published:** 2026-02-09

**Authors:** Zuzanna Borchert, Tomasz Klimczuk, Michał J. Winiarski

**Affiliations:** Faculty of Applied Physics and Mathematics and Advanced Materials Center, 49557 Gdansk University of Technology, Narutowicza 11/12, Gdansk 80-233, Poland

## Abstract

A sample of the cubic Laves phase (*Strukturbericht* C-15) NaAg_2_ was synthesized by prolonged low-temperature annealing (300°C, 3 months) of elemental sodium and silver. Heat capacity measurements were performed on the resulting polycrystalline sample. The compound was found to exhibit metallic properties and no superconducting transition was observed down to *T* = 2 K. The crystal structure of the compound hosts a [Ag_4_]^2–^ pyrochlore network. Density functional theory-based ab initio electronic structure calculations were carried out for NaAg_2_. Based on these, the contribution of [Ag_4_]^2–^ fragment orbitals, the connection of the electronic band structure to the simple four-orbital nearest-neighbor tight binding model, and the importance of higher-order neighbor interactions are discussed.

## Graphical abstract

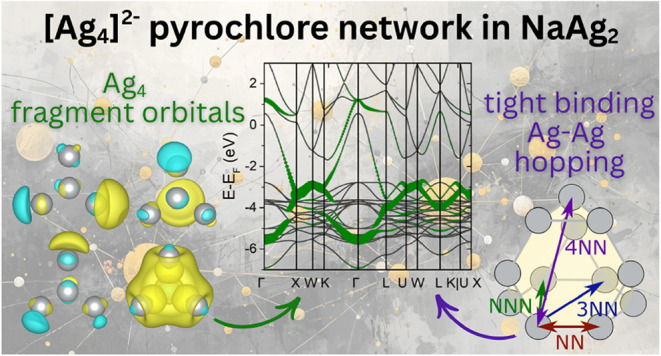

## Introduction

The pyrochlore lattice, a three-dimensional network of corner-sharing tetrahedra, has recently attracted considerable attention as a promising host for exotic magnetism, correlated electron behavior, and nontrivial band topology.^1–8^ The pyrochlore network can be considered a three-dimensional analog of the 2D kagome lattice^9^

The primitive cell of a pyrochlore lattice contains four atoms. A nearest-neighbor (NN) tight binding model constructed with one orbital per atom yields the characteristic electronic band structure (″pyrochlore bands″), shown in Figure 1. A notable feature of the model is the presence of two degenerate flat (nondispersive) bands spanning the whole Brillouin zone (shown in purple and orange in Figure 1). Additionally, the remaining two bands have no dispersions along the X-W path, show saddle points at the L point, and feature a symmetry-protected band crossing at the W and X points.

**Figure 1:**
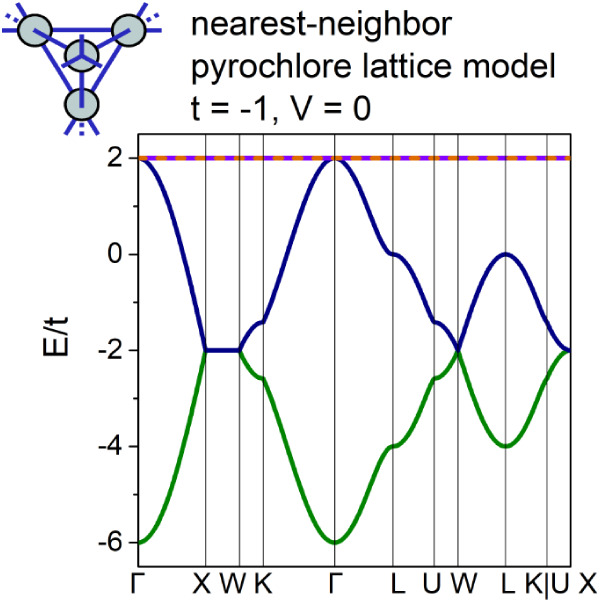
Band structure of a nearest-neighbor (NN) tight binding model of the pyrochlore lattice. The schematic depiction in the top-left corner shows the basic unit of the network (a tetrahedron) and its connectivity. A doubly degenerate topological flat band^8^ is seen at *E*/*t* = 2. Two saddle points found at the L point at *E*/*t* = −4 and 0, result in van Hove singularities of the density of states (DOS). A symmetry protected band crossing is found at X and W points.

Pyrochlore flat band dispersions result from a destructive interference of the electron wave functions and not from lack of orbital overlap.^10^ In the pyrochlore lattice the orbitals interact strongly, but the geometrical frustration prevents electron propagation. Electron wave functions are thus constrained in real space within “compact localized states”.^11^ A similar frustration is observed in other types of lattices, notably the two-dimensional kagome network.^8^

In flat electronic bands the electron–electron interactions are not efficiently screened, leading to many-body effects dominating over the kinetic energy and resulting in exotic non-Fermi liquid behavior.^10,12^ Flat bands at Fermi level can also drive complex magnetism^13^ and are predicted to enhance superconducting critical temperature.^14^

The pyrochlore network is found in several types of crystal structures^15,16^ the most obvious being the pyrochlore type^17^ with the general formula A_2_B_2_X_7_, where two interpenetrating lattices are found, formed by A and B atoms occupying the 16*c* and 16*d* Wyckoff sites of the cubic (space group *Fd*-3*m*) cell. The pyrochlore network is also found in AB_2_X_4_ spinels (B atom network) and in cubic Laves phases (*Strukturbericht* C15, MgCu_2_-type). The latter group is the most common structure type among binary intermetallic compounds.^18^ Cubic Laves phases are reported to display a large variety of interesting properties, including magnetism (both itinerant and local moment), strong magnetocaloric effect^19–22^ superconductivity^6,23–26^ and charge density waves.^27^ In real materials, the pyrochlore structure is never found isolated, and the presence of surrounding sublattices introduces further interactions that can strongly perturb the idealized pyrochlore behavior.^8^ Thus, the band structure depends strongly on orbital interactions between the pyrochlore network and its neighbors within the crystal structure, making chemical bonding analysis useful in understanding the structure–property relationship and in designing new materials with desired properties.^8,28^

In this study we synthesized microcrystalline samples of the cubic Laves phase NaAg_2_ and measured its heat capacity. The crystal structure of the compound was first described by Kienast, Verma, and Klemm in 1961,^29^ but its physical properties were not reported. Our analysis of the results of high-throughput *ab initio* calculations on NaAg_2_ published in the Materials Project (mp-30352)^30,31^ and AFLOW databases (aflow:94289761b4a9021a)^32^ revealed band structure features consistent with a perturbed four orbital pyrochlore model. Such a simple band structure near the Fermi level is uncommon in pyrochlore lattice materials (especially intermetallics), since *p*/*d* orbital degeneracy usually complicates the electronic picture.^5,8^ We performed electronic structure calculations using density functional theory (DFT) and studied the effects of individual interatomic interactions on the band structure using a series of tight binding (TB) models. The interesting feature of the band structure of NaAg_2_ is that it can be understood in terms of a simple four-orbital TB model of a pyrochlore lattice with longer-range interactions included. Our results can be useful in understanding and designing pyrochlore lattice materials.

## Materials and Methods

Polycrystalline samples of NaAg_2_ were synthesized by reacting stoichiometric amounts of metallic sodium (99.8%, Onyxmet, Poland) and silver powder (99.9%, Mennica-Metale, Poland). The reagents were loaded into a pressing die and formed into a 10 mm diameter pellet. The pellet was then put into a niobium crucible, which was subsequently crimp-sealed to prevent Na vaporization losses. The whole procedure was performed inside an Ar-filled glovebox. The tantalum crucible was then sealed inside an evacuated and Ar-backfilled fused quartz tube without exposing the contents to air.

Since the NaAg_2_ phase was reported to possibly undergo a peritectic decomposition at above 322°C^29^ and due to low melting point of sodium (97.8°C),^33^ the sample was slowly heated (2°C/h) from room temperature to 300°C in a lab oven with fan convection and then soaked for 3 months, followed by slow cooling to 100°C. The resulting pellet had a dark color on the outside and a silver luster inside and was brittle. When exposed to air, the powdered sample quickly tarnished and decomposed. Scanning electron microscopy imaging (see Figure 2) revealed agglomerations of sub-1 mm, well-defined octahedral microcrystallites.

**Figure 2:**
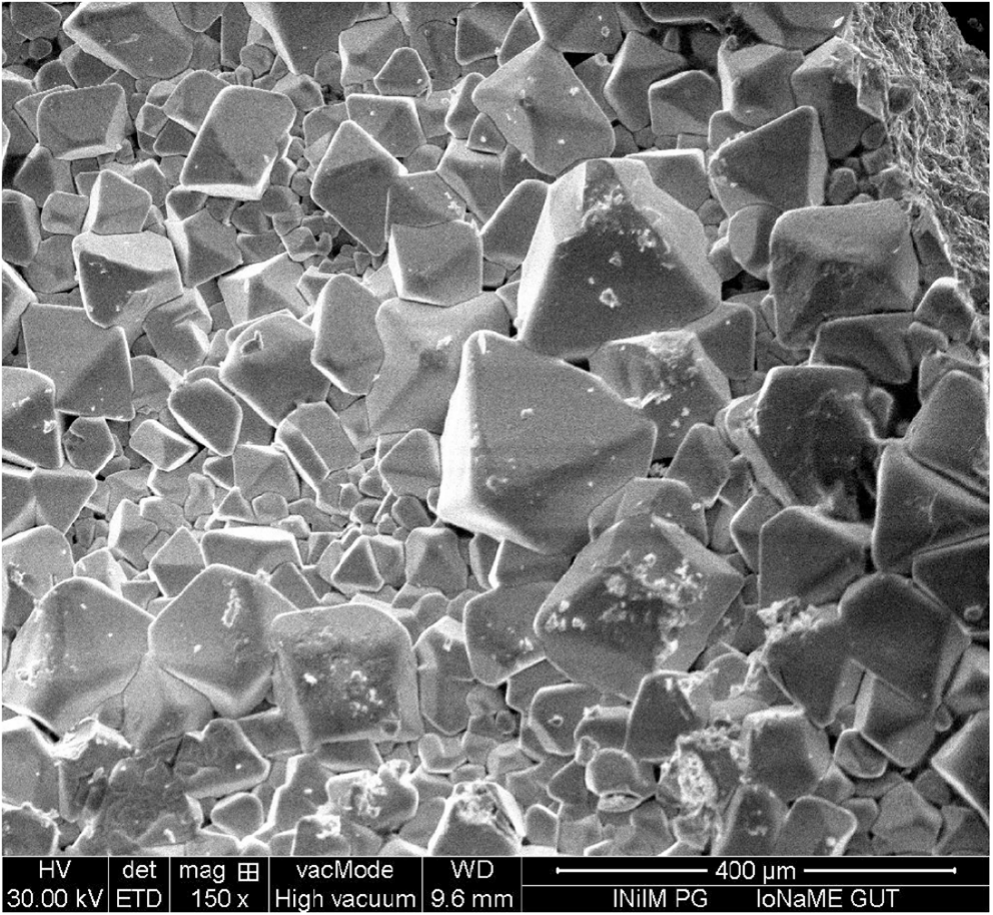
Scanning electron microscopy image of a NaAg_2_ sample obtained after the synthesis procedure. The cubic Laves phase forms octahedral microcrystallites.

Phase composition of the sample was characterized by means of powder X-ray diffraction (pXRD) using a Bruker D2 Phaser instrument with Cu Kα source and a LYNXEYE XE-T position-sensitive detector. To protect the sample from reaction with air and moisture, it was mixed with a small amount of Apiezon N grease. Such prepared sample was stable enough to allow 7 min long measurement, but prolonged exposure to air without the Apiezon N cover resulted in a complete decomposition (see Supporting Information Figure S1). The Bruker TOPAS ver. 6 software used to analyze the pXRD patterns and the Rietveld method was employed to refine unit cell parameters and estimate the phase content.

Heat capacity measurements were carried out using a Quantum Design Evercool II Physical Properties Measurement System (PPMS) and employing the standard semiadiabatic short pulse method. An attempt to measure sample magnetization using the vibrating sample magnetometer (VSM) option of the PPMS was unsuccessful due to the sample’s weak magnetic response.

Density functional theory (DFT) calculations of the electronic structure were performed using the Quantum ESPRESSO package^34–36^ and the Projector-Augmented Wave (PAW) method.^37–39^ The Perdew–Burke–Ernzerhof Generalized Gradient Approximation (PBE GGA)^40^ was employed and the scalar-relativistic PAW sets were taken from the PSlibrary (Na.pbe-spn-kjpaw_psl.1.0.0.UPF, Ag.pbe-n-kjpaw_psl.1.0.0.UPF).^41^ The wave function and charge density cutoff values were set to 60 and 600 Ry, respectively. Self-consistent field (SCF) calculations were completed on a 7 × 7 × 7 Monkhorst–Pack *k*-point grid. For non-SCF calculation of density of states (DOS) and Fermi surfaces a 10 × 10 × 10 grid was used.

The LOBSTER v. 5.1.1 code^42,43^ was employed to project the results of plane-wave DFT calculations onto a local basis and study the fragment orbital-projected band structure. The standard LOBSTER Bunge-Barriento*sec*-Bunge basis set^44^ was used with basis functions 4*d* and 5*s* for Ag and 2*s*, 2*p*, and 3*s* for Na. The results were postprocessed using the LOPOSTER program.^45^

Tight-binding models were constructed using the PyBinding package.^46^

## Results and Discussion

Results of pXRD measurements and Rietveld refinement are shown in Figure 3a. The sample was found to contain NaAg_2_ along with approximately 5 wt % metallic silver. The latter is likely a product of partial decomposition of the main phase (see Figure S1 in the Supporting Information), although it may also originate from residual unreacted Ag powder. The refined unit cell parameter *a* = 7.943(3) Å is in good agreement with previous reports (7.923 Å^29^ or 7.95 Å^47^). The lattice parameter is also similar to the one of the isostructural NaAu_2_ (7.79–7.85 Å).^48,49^ Besides NaAg_2_, no other alkaline metal–silver Laves phases were reported to date.

**Figure 3:**
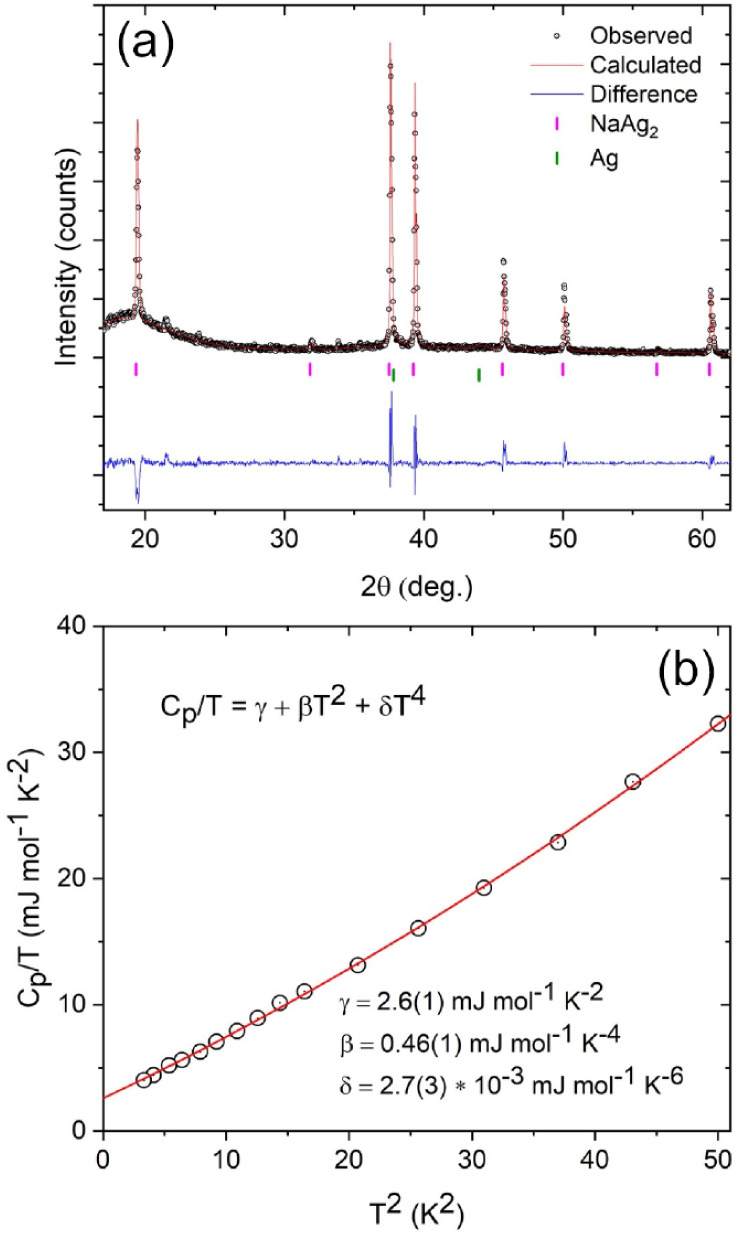
(a) Powder X-ray diffraction pattern of NaAg_2_ sample (black points) with a Rietveld fit (red line). Blue line shows the difference between the measured and calculated intensities. Pink and green ticks mark the expected positions of Bragg reflections for NaAg_2_ and elemental Ag, respectively. (b) Low-temperature heat capacity of NaAg_2_ (black circles). Red line shows the fit to the experimental data.

The measured low-temperature heat capacity of NaAg_2_ is shown in Figure 3b. At *T* = 230 K the heat capacity is close to 70 J mol^–1^ K^–1^, slightly below the Dulong-Petit limit 3*nR* = 74.8 J mol^–1^ K^–1^ (see Figure S2 in the Supporting Information). A fit to the low-temperature heat capacity data (Figure 3b) using eq 1.

1 $$\frac{C_{p}}{T}=\gamma +\beta T^{2}+\delta T^{4}$$

where γ is the Sommerfeld coefficient of the electronic heat capacity, and β and δ are the coefficients of the low-temperature expansion of the Debye model, yields γ = 2.6(1) mJ mol^–1^ K^–2^, β = 0.46(1) mJ mol^–1^ K^–4^ and δ = 2.7(3) × 10^–3^ mJ mol^–1^ K^–6^. Taking the β value, the Debye temperature was calculated using eq 2 yielding θ_*D*_ = 233 K.

2 $$\theta_{D}=\sqrt[{3}]{\frac{12\pi^{4}}{5\beta }nR}$$

The calculated band structure and density of states of NaAg_2_ are shown in Figure 4. Three bands are found to cross the Fermi level as seen in Figure 4a, resulting in the Fermi surface depicted in Figure 5.

**Figure 4:**
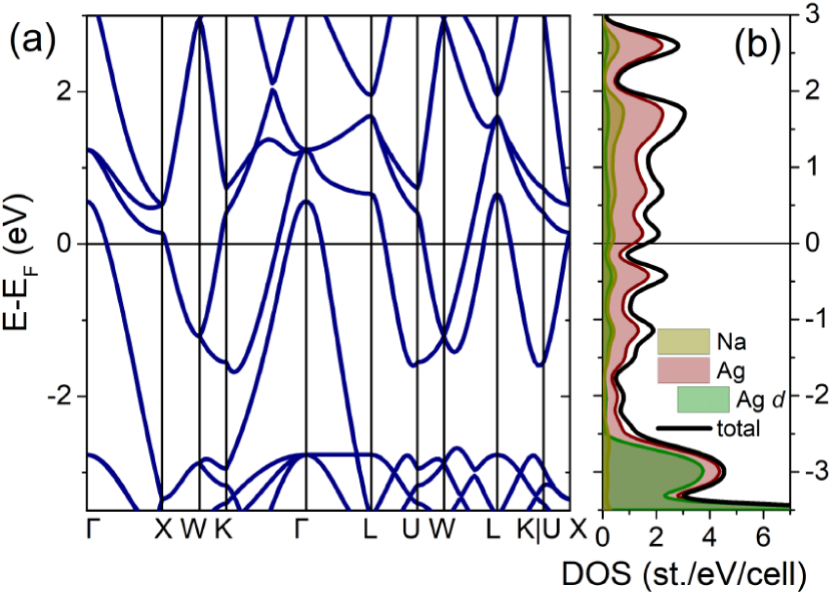
Calculated band structure (a) and density of states (b) of NaAg_2_. High symmetry points of the Brillouin zone are shown in Figure 5.

**Figure 5:**
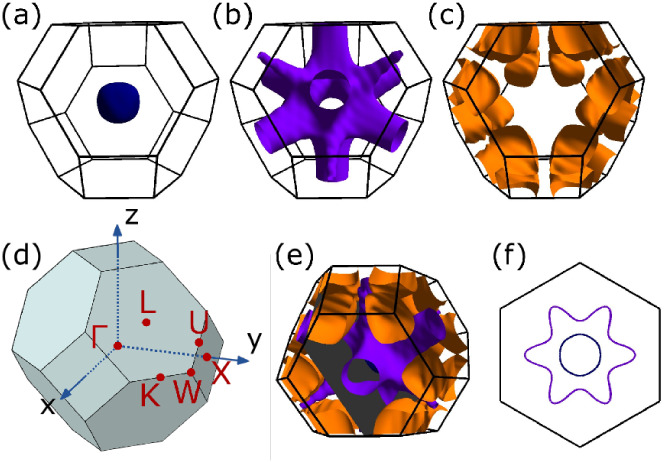
Fermi surface (Fs) of NaAg_2_. Panels (a–c) show the three individual FS branches. High symmetry points of the BZ are shown in panel (d). Panels (e) and (f) show all three FS branches and a section perpendicular to the [111] direction, respectively. The section plane is shown in (e) in gray.

The calculated density of states at the Fermi level *DOS*(*E*_*F*_) = 1.7 states/eV/cell can be used to calculate the Sommerfeld coefficient using eq 3.

3 $$\gamma_{calc}=\frac{\pi^{2}}{3}{k_{B}}^{2}DOS\left(E_{F}\right)$$

This yields γ_*calc*_ = 2.0 mJ mol^–1^K^–2^. Since in a material the density of states is renormalized by electron interactions, most notably the electron–phonon coupling (EPC), the comparison of the DFT-calculated and experimentally measured value of γ yields the EPC coefficient λ_*ep*_ = γ/γ_*calc*_ – 1, yielding λ_*ep*_ = 0.30. This calculation assumes that the heat capacity sample is free of the Ag impurity. If, instead, we take the reported value of the Sommerfeld coefficient for silver^50^ γ_Ag_ = 0.64 mJ mol^–1^K^–2^ and assume the 5 wt % Ag content estimated from the analysis of XRD pattern, the impurity-corrected value $\gamma_{{\mathrm{NaAg}}_{2}}=2.7\mathrm{mJ}{\mathrm{mol}}^{-1}K^{-2}$ = 2.7 mJ mol^−1^K^−2^, yielding λ_*ep*_ = 0.35.

Taking the estimated λ_*ep*_ and θ_*D*_ = 233 K and assuming the typical value of the Coulomb pseudopotential μ* = 0.13 the expected superconducting critical temperature *T*_*c*_ can be estimated using the McMillan formula^51^ (eq 4):

4 $$T_{c}=\frac{\theta_{D}}{1.45}\exp\left(\frac{-1.04\left(\lambda_{ep}+1\right)}{\lambda_{ep}-\mu^{*}\left(1+0.62\lambda_{ep}\right)}\right)$$

This yields *T*_*c*_ = 15 mK for λ_*ep*_ = 0.30 and *T*_*c*_ = 106 mK for λ_*ep*_ = 0.35, consistent with lack of superconducting transition down to *T* = 2 K in heat capacity (Figure 3b).

Figure 6 shows the nearest-neighbor crystal orbital Hamilton population (COHP) and crystal orbital bond index (COBI) for nearest Ag–Ag atoms pairs and [Ag_4_] fragment–Na atom interaction. The integrated COBI (ICOBI) for nearest neighbor (NN) Ag–Ag pairs is 0.066, for next-nearest neighbor (NNN): 0.0074, and for third and fourth NN (3NN and 4NN): 0.0015 and 0.0010, respectively. Integration of −COHP yields ICOHP = 0.28, 0.009, 0.003, and 0.002 for NN, NNN, 3NN, and 4NN, respectively.

**Figure 6:**
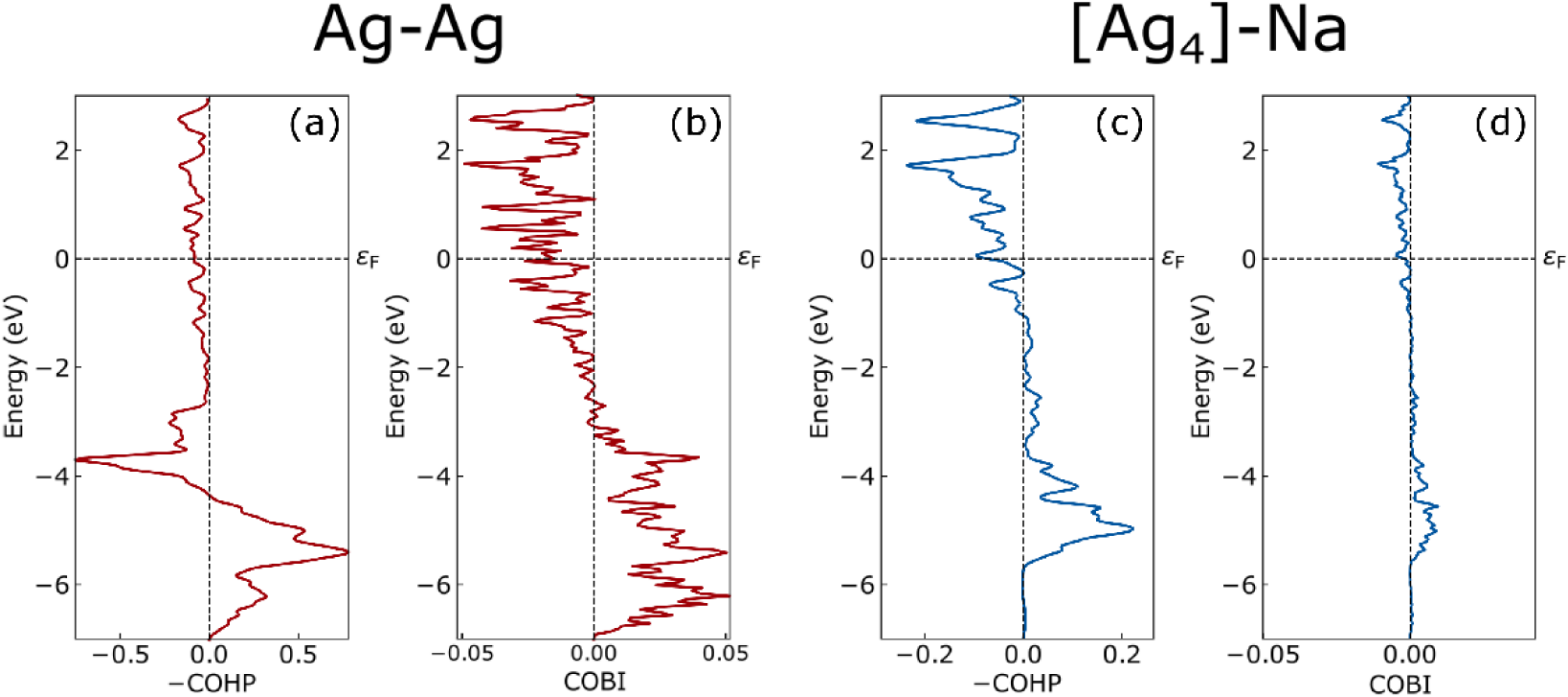
Plots of −COHP­(E) and COBI­(E) between NN Ag atoms (a, b) and between the [Ag_4_] fragment and Na atoms (c,d). Atomic orbital contributions to the Ag–Ag COHP is shown in Figure 7f.

The ICOBI for NN [Ag_4_]–Na interaction is 0.030 and ICOHP = 0.28. This shows that while direct Ag–Ag interactions beyond the NN and NNN range are insignificant, Na atoms provide an indirect bonding pathway between distant Ag neighbors, reminiscent of the magnetic superexchange (or supersuperexchange) mechanism, where a intermediary atom can enable strong coupling between spatially separated magnetic centers.^52,53^

To better understand the connection between chemical bonding and the band structure of NaAg_2_, the fragment orbital (FO) analysis was performed using the linear combination of fragment orbitals (LCFO) method.^43^ Four FOs were found to form the bands around the Fermi level (with 3 crossing the *E*_*F*_), labeled as 2a_1_, 10t_2_, 11t_2_, and 12t_2_. FO wave function isosurface plots and FO-projected band structures are shown in Figure 7.

**Figure 7:**
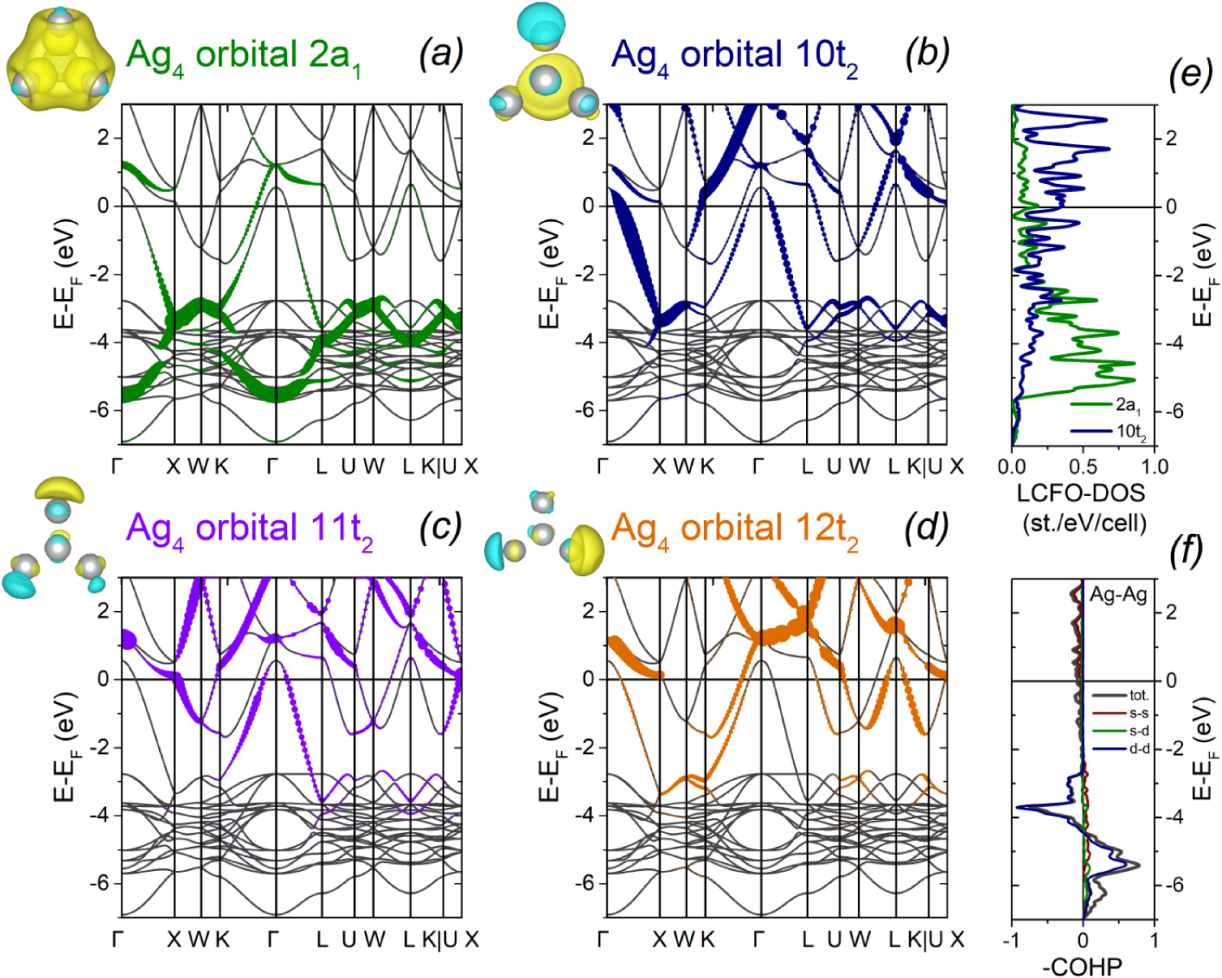
Band structure of NaAg_2_ with the contribution of four frontier orbitals shown using the line width, as derived using the LCFO method. Panel (a) shows the contribution of the nondegenerate 2a_1_, and panels (b–d) the triply degenerate 10/11/12t_2_ FOs. Contribution to the DOS of 2a_1_ and 10t_2_ (LCFO DOS curves of 10t_2_, 11t_2_, and 12t_2_ are identical) is plotted in panel (e). Panel (f) shows orbital contributions to the Ag–Ag COHP. For the molecular orbital formation energy matrix see Figure S3 in the Supporting Information.

While there is no obvious flat band in the band structure of NaAg_2_, one can observe a similarity with tight-binding (TB) model bands. Notably, at the X point, 3 eV below *E*_*F*_ a symmetry-protected crossing of two bands is seen, with a dominant contribution of 2a_1_ and 10t_2_ FOs (Figure 7a,b). The remaining two FOs contribute to two relatively weakly dispersive bands (Figure 7c,d)

To better understand the source of deviation from the simple pyrochlore NN TB band structure, we analyzed the effects of higher-order neighbor interactions (Figure 8) in the tight binding pyrochlore model. The inclusion of NNN hopping results in the flat band attaining dispersion within most of the BZ (Figure 8a). Notably, however, all four bands remain flat along the X-W line with second- and third-nearest neighbor interactions included (Figure 8a,b) and the dispersion appear only when fourth-NN are included (Figure 8c).

**Figure 8:**
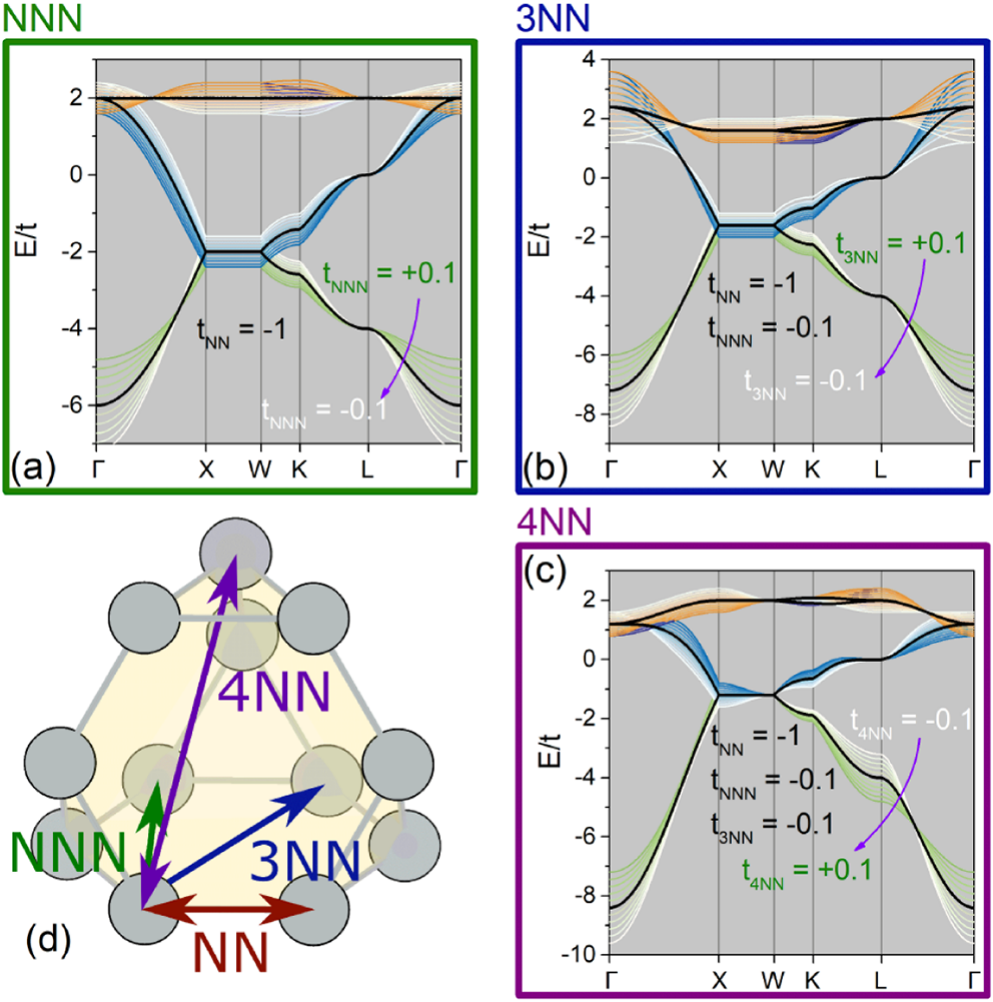
TB models of the four orbital pyrochlore lattice with next-nearest (a), third-nearest (b), and fourth-nearest neighbor interactions included (c). In panels (a–c) traces corresponding to different values of the hopping parameter for the highest included neighbor order are shown with successively fading colors. Panel (d) visualizes the nearest-, next-nearest and higher order interactions.

The common feature of NN, NNN, and 3NN interactions is that they connect atoms lying within a common hexagon (Figure 8d). The 4NN link is the lowest order connecting two neighboring hexagons. The two compact localized states associated with the doubly degenerate topological flat band (TFB) in the NN TB pyrochlore model are constrained to the hexagonal rings of the network. Thus, interactions that allow hopping between these hexagonal rings allow electrons to propagate, resulting in a finite dispersion.

For an isolated pyrochlore network the 4NN interactions could be expected to be very weak due to the large spatial separation. However, in NaAg_2_ the Na sublattice promotes the interaction. The doubly degenerate TFB is affected strongly, as the two [Ag_4_] FOs efficiently overlap with Na *s* atomic orbitals.

## Conclusions

The cubic Laves phase NaAg_2_ was found to show metallic properties as evidenced by a silver luster, the finite value of the Sommerfeld coefficient, γ, and the calculated band structure showing no band gap. No superconducting transition was observed down to *T* = 2 K in accordance with the value of *T*_*c*_ = 15−106 mK estimated using the McMillan equation.

The band structures calculated for NaAg_2_ showed some resemblance to the simple four-orbital tight binding model of the pyrochlore lattice, although the expected doubly degenerate topological flat band has a finite dispersion that could be accounted for by including second- and higher order neighbor hopping. Interestingly, we showed that the flat fragment of the band structure between X and W points attains dispersion only after fourth-nearest neighbor interactions are included, which connect two different hexagonal loops of the pyrochlore network.

Our results demonstrate the effects of individual atomic interactions on the details of the pyrochlore band structure.

## Supplementary Material



## Notes

Result of pXRD study of sample decomposition in air; heat capacity of NaAg_2_ in a wide temperature range; integrated molecular orbital formation energy matrix for the [Ag_4_] fragment (PDF)

## Abbrevations

3NN: third-nearest neighbor
4NN: fourth-nearest neighbor
BZ: Brillouin zone
DFT: density functional theory
DOS: density of states
EPC: electron–phonon coupling
FO: fragment orbital
LCFO: linear combination of fragment orbitals
NN: nearest neighbor
NNN: next-nearest neighbor
pXRD: powder X-ray diffraction
SCF: self-consistent field
TB: tight binding
