# Supplementary material for: Electronic Structure and Thermal Properties of a Cubic Laves Compound NaAg2 Featuring a [Ag4]2– Pyrochlore Network
Source: Inorg Chem. 2026 Feb 9;65(7):4184–90. doi: 10.1021/acs.inorgchem.5c05773 (PMC13298885; doi:10.1021/acs.inorgchem.5c05773)
Supplement: Supplementary file 1 [file ic5c05773_si_001.pdf]

Electronic Structure and Thermal Properties of a Cubic Laves Compound $NaAg_2$ Featuring a $[Ag_4]^{2-}$ Pyrochlore Network
SUPPLEMENTARY MATERIAL

Zuzanna Borchert[1], Tomasz Klimczuk[1], and Michał J. Winiarski*[1]

[1]Faculty of Applied Physics and Mathematics and Advanced Materials Center, Gdansk University of Technology, Narutowicza 11/12, 80-233 Gdansk, Poland

*Email: michal.winiarski@pg.edu.pl

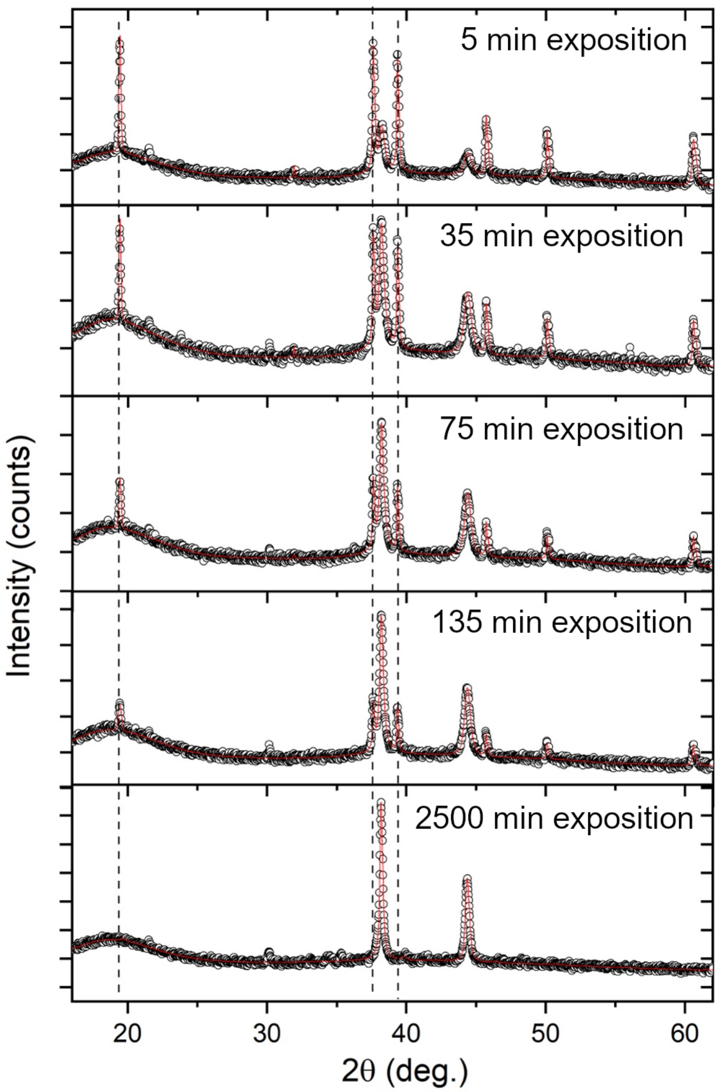

Figure S1: Series of XRD patterns collected on a air-exposed ground $NaAg_2$ sample (with 5 min, 35 min, 75 min, 135 min and 2500 min exposition time) showing a progressive decomposition of the compound (dashed lines indicate positions of three prominent peaks of the Laves phase). After 2500 min exposure the only observable peaks correspond to elemental Ag (FCC structure).

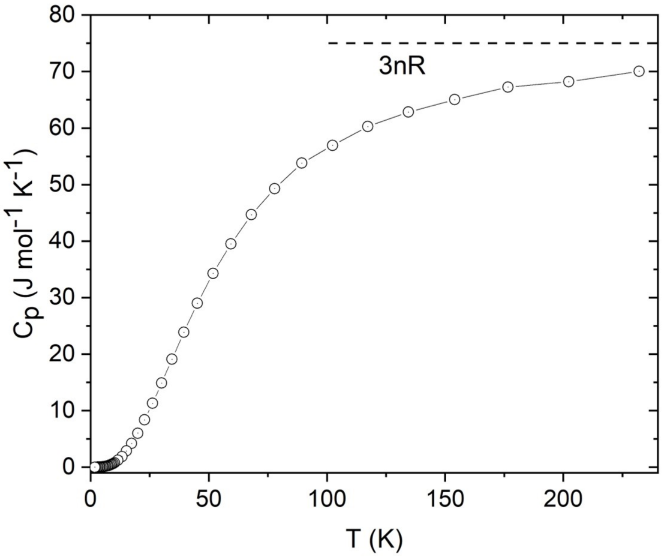

Figure S2: Temperature-dependent heat capacity of $NaAg_2$ (black circles). The Dulong-Petit law limit ($3nR$) is shown with a dashed line.

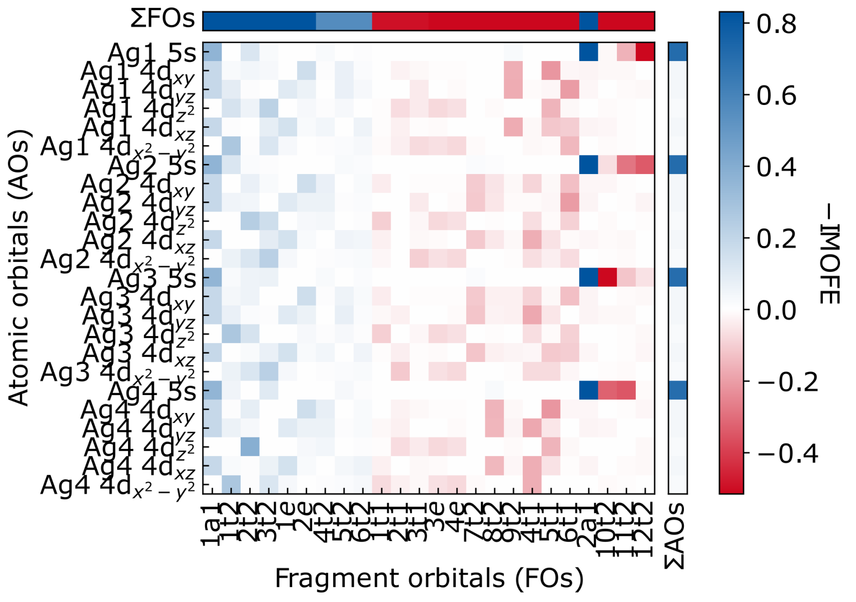

Figure S3: Integrated molecular orbital formation energy matrix for the $[Ag_4]$ fragment.
